# Incidental 68Ga-DOTATATE uptake in the pancreatic head: A case report and a unique opportunity to improve clinical care

**DOI:** 10.1097/MD.0000000000020197

**Published:** 2020-05-29

**Authors:** Rahul Lakhotia, Sakshi Jhawar, Ashkan A. Malayeri, Corina Millo, Jaydira Del Rivero, Mark A. Ahlman

**Affiliations:** a Medical Oncology Service, Center for Cancer Research, National Cancer Institute, Clinical Center; b Eunice Kennedy Shriver National Institute of Child Health and Human Development; c Radiology and Imaging Sciences, Clinical Center; d Pediatric Oncology Branch, Center for Cancer Research, National Cancer Institute, Clinical Center, National Institutes of Health, Bethesda, MD 20892.

**Keywords:** 68Ga-DOTATATE, neuroendocrine tumors, pNET, somatostatin receptor-based imaging, uncinate process

## Abstract

**Rationale:**

Neuroendocrine tumors (NETs) are neoplasms that can arise from the neuroendocrine cells distributed widely throughout the body. Majority of NETs overexpress somatostatin receptors (SSTR) on their cell surface. This biologic characteristic is exploited by SSTR-based imaging such as ^111^In octreotide scintigraphy and ^68^Ga DOTATATE positron emission tomography (PET)/computed tomography (CT), which are considered standard for initial evaluation of NETs. Although highly sensitive and specific, recent reports demonstrate a concerning incidence of “false-positive” physiologic uptake of these tracers in the pancreatic head – a common site of neuroendocrine tumor (NET) involvement. We present false positive uptake on ^68^Ga DOTATATE PET/CT along with false positive CT findings. Role of other imaging modalities is discussed.

**Patient concerns:**

A 78-year-old woman presented with a year-long history of diarrhea.

**Diagnosis:**

Serum vasoactive intestinal peptide (VIP) levels were slightly elevated at 134.2 pg/mL (normal <75 pg/mL). CT showed a mildly enhancing 2.5 cm × 1.8 cm × 2.8 cm area in the pancreatic uncinate process which corresponded to focal uptake with ^68^Ga DOTATATE PET/CT. A presumptive diagnosis of pancreatic NET (vipoma) was made, and the patient was scheduled to undergo Whipple's surgery.

**Interventions:**

She sought a second opinion and a subsequent magnetic resonance imaging (MRI) showed no lesion and the patient's surgery was deferred. Thereafter, her VIP levels spontaneously normalized. Endoscopic ultrasound (EUS) with fine needle aspiration cytology of the uncinate process showed normal pancreatic acini with no evidence of NET.

**Outcomes:**

Patient is currently pursuing workup for alternative etiologies for chronic diarrhea.

**Lessons:**

Conspicuous physiological uptake has been reported in the pancreatic head on 16% to 70% of ^68^Ga DOTATATE or ^68^Ga DOTANOC PET/CT scans, and 26% of the ^111^In octreotide scintigraphy scans. Image-based quantitative attempts to distinguish physiologic from pathologic uptake using SUV_max_ have rendered mixed results. When evaluating SSTR-based imaging uptake in the pancreatic head, patients can benefit from a higher index of suspicion of false positive uptake. Such cases require additional confirmation by MRI or EUS. Interestingly, the patient described also had mild contrast enhancement on CT, but without an MRI correlate. Because of potential morbidity and mortality related to false positive uptake, a systematic review with evidence-based recommendations for imaging may benefit patient care.

## 1 Introduction

Neuroendocrine tumors (NETs) are epithelial neoplasms that can arise from the neuroendocrine cells distributed widely throughout the body. The majority of NETs overexpress somatostatin receptors (SSTR) on their cell surface. This biologic characteristic is exploited in the somatostatin receptor-based imaging techniques such as ^111^In octreotide scintigraphy and ^68^Ga DOTATATE positron emission tomography (PET). These modalities are currently considered standard for the initial evaluation of suspected neuroendocrine tumors. Although highly sensitive and specific for NETs, recent reports demonstrate a concerning incidence of “false-positive” physiologic uptake of these tracers in the pancreatic head – a confounding common site of neuroendocrine tumor (NET) involvement. This report is intended to further highlight a persistent concerning issue and to bring to attention the inadequacy of cross-sectional imaging with computed tomography (CT) only in this situation. Potential approaches for when such a finding occurs is discussed.

## 2 Case report

**Figure 1:**
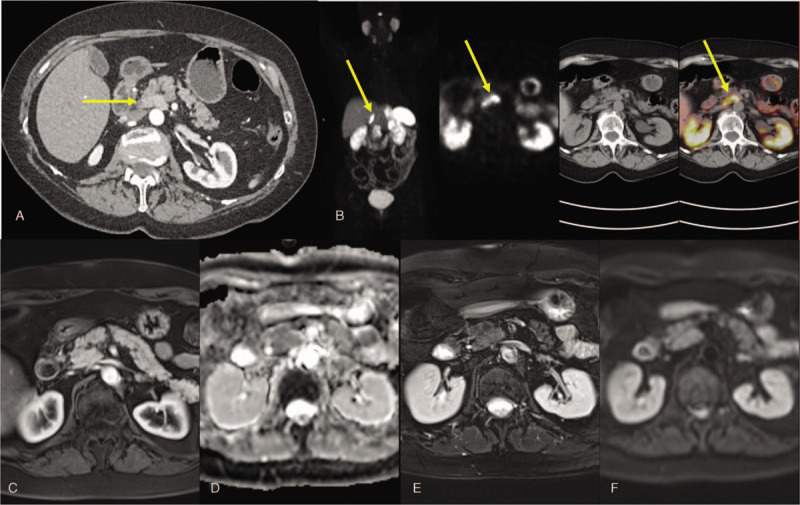
(A) Computed tomography (CT) scan showing mild enhancement in the pancreatic uncinate process. (B) ^68^Ga DOTATATE positron emission tomography (PET)/CT imaging demonstrating uptake in the pancreatic uncinate process. (C–F) MRI with no corresponding anatomic abnormality on (C) T1 post-contrast, (D) apparent diffusion coefficient (ADC), (E) T2-weighted fat suppressed, and (F) diffusion-weighted imaging (DWI).

A 78-year-old woman presented to clinic with a year-long history of diarrhea. Patient had 4 to 5 loose, greasy stools per day. She did not report any blood in stools, weight loss, dyspnea, facial flushing, skin rash, nausea, or vomiting. Patient had previously tried bland diet, cholestyramine, and Pepto-Bismol without any improvement in symptoms. Past medical history was significant for breast cancer diagnosed 5 years ago and treated with lumpectomy, radiation, and anastrozole; hypertension well-controlled on Valsartan; and hypothyroidism treated with levothyroxine replacement. Physical examination did not elicit any abdominal tenderness, guarding, or rigidity. Serum vasoactive intestinal peptide levels were slightly elevated at 134.2 pg/mL (normal <75 pg/mL). CT showed a mildly enhancing 2.5 cm × 1.8 cm × 2.8 cm area in the pancreatic uncinate process which corresponded to mild to moderate uptake with ^68^Ga DOTATATE PET-CT imaging (Fig. 1A–B). Based on these findings, a presumptive diagnosis of pancreatic NET (vipoma) was made, with subsequent scheduling for a Whipple's surgery. The patient sought a second opinion at our institution as part of an IRB-approved trial, and signed informed consent.

A subsequent magnetic resonance imaging (MRI) demonstrated no lesion in the uncinate process and the patient's surgery was deferred (Fig. 1C–F). On a future measurement, after adequate patient preparation, her vasoactive intestinal peptide (VIP) levels were normal (<50 pg/mL, reference range <75 pg/mL). Endoscopic ultrasound (EUS) with fine needle aspiration cytology of the uncinate process showed no evidence of involvement with NET. Patient is currently pursuing workup for alternative etiologies for chronic diarrhea.

## 3 Discussion

The case presented here highlights the importance of differentiating physiologic uncinate process uptake from pathological uptake on SSTR-based imaging. A unique aspect of this case was that the CT scan also demonstrated enhancement in the pancreatic head region suggesting that better cross-sectional imaging modalities are required to characterize the uptake in pancreatic head on SSTR-based imaging. Here we will discuss the available evidence in the literature with suggested clinical approaches when such a finding occurs.

In 2016, Ramos, et al reported 2 cases of clinically suspected NET who underwent ^68^Ga DOTANOC PET-CT with presumptively abnormal uptake in the pancreatic uncinate process,^[1]^ without a corresponding anatomical abnormality on EUS. Other case series have corroborated these findings with conspicuous physiological uptake in the pancreatic head reported to be present on 16% to 70% of ^68^Ga DOTATATE or ^68^Ga DOTANOC PET-CT scans (Table 1).^[2–4]^ Brabender et al found physiologic uptake in the region of the pancreatic head in 26% (46/178) patients who underwent ^111^In octreotide scintigraphy. Interestingly, the incidence of physiological uptake was 50% (10/20) in patients on antidiabetic drugs.^[4]^ The abundance of pancreatic polypeptide (PP) cells in the uncinate process of the pancreatic head (90% of all pancreatic PP cells) is thought to be the reason for this physiologic uptake, due to SSTR subtypes 1 to 4 cell surface expression.^[4]^

**Table 1:**
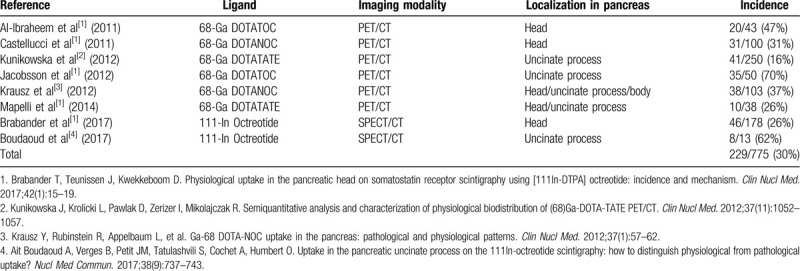
Reported incidence of physiological uptake in the pancreas.

Image-based quantitative attempts to distinguish physiologic from pathologic uncinated process uptake have rendered mixed results. Compared to tumors, physiologic uptake generally shows lower PET standardized uptake value (SUV) and is less focal in appearance. In 390 ^68^Ga DOTATOC scans performed on patients with confirmed or suspected SSTR expressing malignancies, Kroiss et al reported a cutoff SUV_max_ of 17.1 to differentiate tumors in the uncinate process with 93.6% specificity and 90.0% sensitivity.^[5]^ However, another study was unable to reach similar performance characteristics based solely on SUV_max_.^[3]^ Importantly, it is well established that SUV is highly dependent on institution and technology-specific methods of image acquisition and reconstruction, which is in part due to the lack of a widely deployed means to standardize SUV quantification among institutions and scanner vendors. Thus, we advise care when attempting to apply SUV cutoffs to a clinical delineation of pathology from physiology. For example, Krausz et al performed synchronous functional (^68^Ga DOTANOC PET-CT) and anatomic (CT or MRI) imaging in 96 patients with pancreatic or nonpancreatic NET, and biochemical suspicion of disease. 24 foci in the pancreas of 21 patients (SUV_max_ range 4.7–35) were considered suspicious for NETs with PET-CT, but no tumor was seen on concurrent anatomic imaging or upon 13-month follow-up with ^68^Ga DOTANOC PET-CT.^[3]^ In another series of 13 patients with isolated uptake in the head of the pancreas on ^111^In octreotide scintigraphy, EUS demonstrated a corresponding pancreatic lesion in only 5 (38%)cases.^[6]^ Among the 8 EUS negative patients, 4 had a biopsy that revealed no pathological cells. With 1 to 4 years of imaging follow-up, none of these patients had any evidence of NET located in the head of the pancreas. For all EUS negative patients, the uptake was located in the uncinate process. The investigators also compared the intensity of this uptake with uptake in the liver at 6 and 30 hours post-tracer injection, and a ratio between the pancreatic uptake and hepatic uptake (L/H ratio) was calculated. At both 6 and 30 hours, L/H ratio in all 8 EUS negative patients was less than 2, whereas it was higher than 2 in 4/5 patients with a corresponding anatomic pancreatic lesion.

Interestingly, in the case presented above, CT scan also showed mild enhancement in the region of pancreatic head further adding to the dilemma. While CT is the most widely used “cross-sectional” imaging method for detection of neuroendocrine tumors of the pancreas, this technique only takes advantage of 1 imaging characteristic of these tumors, that is, hyperenhancement.^[7]^ In the meantime, MRI is a versatile technique with superior soft tissue characterization capabilities compared to CT. This detailed soft tissue characterization is achieved by applying different MRI techniques (sequences) in 1 session, tailored to the expected imaging characteristics of the lesion. For example, pancreatic NETs are generally more T2 hyperintense compared to the background normal pancreas. These lesions also demonstrate hyperenhancement and restricted diffusivity of the water molecules due to increased angiogenesis and higher cellular density, which can be detected on dynamic contrast-enhanced MRI and diffusion-weighted imaging techniques, respectively.^[8,9]^ These capabilities have led some researchers to conclude that MRI is a reasonable alternative to EUS for detection of pancreatic NETs.^[10]^ At our institution, we have adopted a comprehensive MRI technique, with and without contrast, as the main cross-sectional imaging modality, complementary to targeted molecular imaging for detection and follow-up of these pancreatic lesions.

In conclusion, when evaluating somatostatin receptor-based molecular imaging (e.g., ^68^Ga-DOTATATE) uptake in the pancreatic head, this report recapitulates a recommendation of a high index of suspicion of false positive uptake, which can help avoid unnecessary invasive procedures (e.g., biopsy, Whipple's procedure). Such cases likely require more extensive confirmation, which ideally includes a comprehensive MRI of the pancreas.

## Author contributions

Rahul Lakhotia - Data curation, Formal analysis, Investigation, Methodology, Visualization, Writing (original draft and review/editing) Sakshi Jhawar - Data curation, Investigation, Methodology, Visualization, Writing (review/editing) Ashkan A. Malayeri - Conceptualization, Investigation, Visualization, Writing - review & editing Corina Millo - Conceptualization, Investigation, Visualization, Writing – review & editing Jaydira Del Rivero - Conceptualization, Investigation, Visualization, Methodology, Resources, Supervision, Writing – review & editing Mark A. Ahlman - Conceptualization, Data curation, Formal analysis, Funding acquisition, Investigation, Methodology, Project administration, Supervision, Validation, Visualization, Writing (original draft and review/editing).
